# Cyclosporine a drug-delivery system for high-risk penetrating keratoplasty: Stabilizing the intraocular immune microenvironment

**DOI:** 10.1371/journal.pone.0196571

**Published:** 2018-05-07

**Authors:** Ting Zhang, Zhiyuan Li, Ting Liu, Suxia Li, Hua Gao, Chao Wei, Weiyun Shi

**Affiliations:** 1 State Key Laboratory Cultivation Base, Shandong Provincial Key Laboratory of Ophthalmology, Shandong Eye Institute, Shandong Academy of Medical Sciences, Qingdao, China; 2 Shandong Eye Hospital, Shandong Eye Institute, Shandong Academy of Medical Sciences, Jinan, China; INSERM, FRANCE

## Abstract

Cyclosporine A (CsA) is an essential medication used to prevent corneal allograft rejection. Our preliminary studies revealed that CsA drug-delivery system (DDS) was more effective in preventing high-risk corneal allograft rejection than topical CsA application. However, the impacts of CsA DDS on the intraocular immune microenvironment were not fully elucidated. In the present study, we investigated the effect of CsA DDS on the cornea allograft, aqueous humor, and iris-ciliary body using a rabbit model of high-risk penetrating keratoplasty. New Zealand white rabbits were divided into four groups: a normal control group, an untreated group, a CsA eye drop group and a CsA DDS group. Graft survival was monitored for 12 weeks, and the therapeutic effects of CsA DDS were evaluated at 3 and 12 weeks after high-risk keratoplasty. In the CsA DDS group, the mean graft survival time was significantly prolonged when compared with the untreated and CsA eye drop groups. At all time-points, Langerhans cell density, inflammatory cell density, and central corneal thickness in the CsA DDS group were much lower(all p < 0.01) than the untreated and CsA eye drop groups, in which their parameters were significantly higher than the normal control group (all p < 0.01). Compared with the untreated and CsA eye drop groups, an implanted CsA DDS markedly decreased the CD11b^+^ and CD8^+^ T cell infiltration in the corneal grafts. CsA DDS treatment also greatly reduced the CD4^+^ T cell density and the expression of interferon-gamma, interleukin-2 (IL-2), IL-6, CD80, and CD86 mRNA both in the corneal graft and iris-ciliary body (all p < 0.01). Moreover, CsA DDS significantly reduced the IL-2 level in aqueous humor (p < 0.01). Taken together, our results suggest that CsA DDS implanted into the anterior chamber create a relative immunosuppressive microenvironment in the corneal graft, iris-ciliary body, and aqueous humor. Stabilizing the intraocular immune microenvironment could partially elucidate the mechanism of CsA DDS in suppressing corneal graft rejection.

## Introduction

Immunological allograft rejection is considered the leading cause of corneal graft failure [1, 2]. However, the exact mechanisms leading to graft rejection have not been fully understood. For the epithelial and stromal rejection, a vascularized cornea may deliver effector cells to the corneal grafts through the classic corneal limbus pathway, while for the endothelial rejection, the leukocytes are likely to be egressed from the iris-ciliary body, which must pass through the anterior chamber before targeting the corneal grafts [3, 4].

Recent studies have shown that the presence of immunosuppressive molecules in aqueous humor, such as transforming growth factor-2 and soluble Fas ligand, together with the immunomodulatory features of the iris-ciliary body pigment epithelium, constitute the immune-privileged property of anterior chamber [5, 6]. The intraocular immunosuppressive climate might influence the development of immune reaction and the long-term graft survival. Thus, more attention should be paid to the anterior chamber microenvironment for a better understanding of corneal graft immune rejection.

Multiple studies have evaluated the good biocompatibility and complete biodegradability of cyclosporine A drug-delivery system (CsA DDS) [7–10], and proved its safety and efficacy in the prognosis of corneal graft rejection [11, 12]. In this study, we focused on the pathological changes in the corneal graft, iris-ciliary body, and aqueous humor during corneal immune rejection, and investigated the influence of CsA DDS on the intraocular microenvironment to shed further light on its mechanism.

## Materials and methods

### Ethics approval

The animal experiment was approved by the Ethics Committee of the Shandong Eye Institute (No. SEIRB-2015-224A12), with all procedures being conducted conforming to the Association for Research in Vision and Ophthalmology (ARVO) statement. All surgeries were performed on one eye of each rabbit under systemic and topical anesthesia. The suffering was minimized, and the animals were sacrificed by aeroembolism.

### Animals

Male New Zealand white rabbits (Kangda Co., Shandong, China), weighing 2.0–2.5 kilograms, were used to establish a model of high-risk penetrating keratoplasty (PKP). They were randomly divided into four groups: a normal control group with no procedure performed (n = 10), an untreated group without CsA treatment after high-risk keratoplasty (n = 18), a CsA eye drop group receiving 1% CsA eye drops (Huabei Pharmaceutical Co., Shijiazhuang, China) four times a day (n = 10) after surgery, and a CsA DDS group with DDS implanted during keratoplasty (n = 18).

### CsA DDS preparation

CsA DDS was prepared as previously described [11, 12]. Polylactide-co-glycolide-co-caprolactone (PGLC) was employed as the carrier (Institute of Chemistry, Chinese Academy of Sciences, Beijing, China). Briefly, 1.0 mg PGLC and 1.0 mg CsA (Novartis, Basel, Switzerland) were dissolved in chloroform. The resultant solution was lyophilized, and compressed into round films (diameter: 2.0 mm, thickness: 0.5 mm) each weighing 2.0 mg and containing 1.0 mg CsA. The fabricated CsA DDS was sterilized with epoxy ethane steam for 24 hours, and then packed into a germ-free tube for use.

### High-risk penetrating keratoplasty

Anesthesia was induced by intramuscular injection of 25 mg/kg ketamine and 25 mg/kg chlorpromazine. Neovascularization was induced by placing a 5–0 silk suture (Mani, Japan) in each quadrant of the cornea as previously described [13]. Within two weeks, the cornea was strongly vascularized with vessels growing at least 4 mm into three or more quadrants. Thereafter, the sutures were removed, and PKP was performed.

A 7.75-mm trephine was used to produce a corneal button graft, and the recipient cornea was prepared similarly with a 7.5-mm biopsy punch. Then, the donor graft was placed centrally into the vascularized bed with 10–0 monofilament nylon sutures (Mani, Japan). A CsA DDS was implanted into the inferior angle of the anterior chamber before the last suture of keratoplasty [13]. During surgery, topical heparin (1000 U/ml) was given to prevent aqueous clotting formation. Ofloxacin eye ointment (0.3%; Santen, Osaka, Japan) was administered at the end of the procedure and once a day for three subsequent days. The eyes in the CsA eye drop group received 1% CsA eye drops four times a day throughout the study.

### Clinical evaluation

The corneal allografts were examined by slit lamp microscopy each day for the first 3 weeks and twice a week thereafter. A rejection index on a scale of 0 to 12 was calculated by combining the scores of three indicators (opacity, edema, and neovascularization) in the allograft, with each indicator scoring on a scale of 0 to 4. A value of the index greater than or equal to 6 was considered as allograft rejection. The scoring criteria used were of minor modifications on the basis of a previous report [13]. For opacity: 0, clear cornea; 1, slight haze; 2, increased haze but the anterior chamber still clear; 3, advanced haze with blurred view of the anterior chamber; 4, opaque cornea with no view of the anterior chamber; for edema: 0, no stromal or epithelial edema; 1, slight stromal edema; 2, diffuse stromal edema; 3, diffuse stromal edema with epithelial microcysts; 4, bullous keratopathy; for neovascularization: 0, no neovascularization at graft-host junction (GHJ); 1, neovascularization at GHJ in one quadrant only; 2, neovascularization at GHJ in two quadrants; 3, neovascularization at GHJ in three quadrants; 4, vascularization at GHJ in all four quadrants. To minimize the inter-observer variability, 10 animals of each group were scored twice by two observers in a masked fashion (TZ, TL), and the mean score was used in further analysis.

In addition, the central corneal thickness (CCT) was measured by anterior segment optical coherence tomography (AS-OCT, Carl Zeiss Meditec, Oberkochen, Germany). The Langerhans cell density, inflammatory cell density, and endothelial cell density were assessed by laser scanning confocal microscopy (HRT-II, Heidelberg, Dossenheim, Germany), on which all animals were fixated on a distance target aligned to enable the full thickness scan of the central cornea. To minimize the inter-observer variability, the cell number in 10 randomly selected photographs from each group was counted twice by both observers in a masked fashion (TZ, TL).

### Histopathological examination

The rabbits were euthanized at 3 or 12 weeks after keratoplasty, and the cornea and iris-cliary body were carefully harvested and embedded in the OCT compound (Sakura, USA). Subsequently, serial sections with a thickness of 6 μm were prepared for immunofluorescence staining.

The slices were fixed in 4% paraformaldehyde for 15 minutes before treated with 0.1% Triton-X 100 for 15 minutes. After blocked with 5% bovine serum albumin at room temperature for one hour, they were incubated with CD4 (ab194998, Abcam), CD11b (101207, Biolegend), and CD8 (ab41323, Abcam) antibodies at 4°C overnight, and then reacted with fluorescent secondary antibody (Abcam) at room temperature for one hour. Finally, stained with DAPI, fluorescence micrographs of the cornea and iris were visualized and photographed by fluorescence microscopy (Nikon, Tokyo, Japan). The amount of CD4^+^ T cells in each micrograph from different groups was counted twice in a masked fashion by two investigators (TZ, TL).

### Quantitative real-time PCR

Total RNA was extracted from the cornea or iris-ciliary body with the TRIzol reagent (Invitrogen, Carlsbad, CA, USA), before reverse-transcribed utilizing the PrimeScript RT Reagent Kit (Takara Bio Inc., Otsu, Japan) according to the manufacturer's protocol. Quantitative real-time PCR (qRT-PCR) was performed in a 7500 real-time PCR machine (Applied Biosystems, Foster City, CA, USA) with the SYBR Green PCR reagent (Takara Bio Inc., Otsu, Japan). GAPDH was taken as an endogenous control, and the primer sequences used are listed in Table 1.

**Table 1:** Primer sequences used for qRT-PCR.

Gene	Orientation	Primer sequence (5' - 3')
GAPDH	Forward	CGCCTGGAGAAAGCTGCTAA
	Reverse	CCCCAGCATCGAAGGTAGAG
IFN-γ	Forward	TCTTGGGTTCTTACGGCTGTTAC
	Reverse	TGTCACTCTCCTCTTTCCAATTCC
IL-2	Forward	AACCTCTGGAGGAAGTGCTTAACTT
	Reverse	CAATGGTAACTGTCTCATCGTATTCA
IL-6	Forward	GAACCTGCAGCAGAAAAACCA
	Reverse	GAGGCAGAGCCCATGAAATTC
CD80	Forward	GCCTGGATGGAAGATGGAGAA
	Reverse	CAGCTCCCCGTATTTGATGAG
CD86	Forward	TCAGCGGATCTATCACATTCTCA
	Reverse	CTGCAATCCAGAGCCTTGGT
FOXP3	Forward	TCCTCCAGAGAGAGGTGGTACAGT
	Reverse	TACAATGCAGCAGGAGCTCTTG

### Enzyme-linked immunosorbent assay (ELISA)

Aqueous humor was collected. The level of interleukin (IL-2) in the aqueous humor was determined using a commercially available ELISA kit (Uscn Life Science, Wuhan, China).

### Statistical analysis

All experiments were performed on at least three occasions, with the data being presented as mean ± standard deviation. The Kaplan-Meier survival method was used to compare the survival of corneal allografts, while the one-way analysis of variance (ANOVA) was employed to make comparisons between groups. A value of p < 0.05 was considered statistically significant.

## Results

### Graft survival time

**Fig 1:**
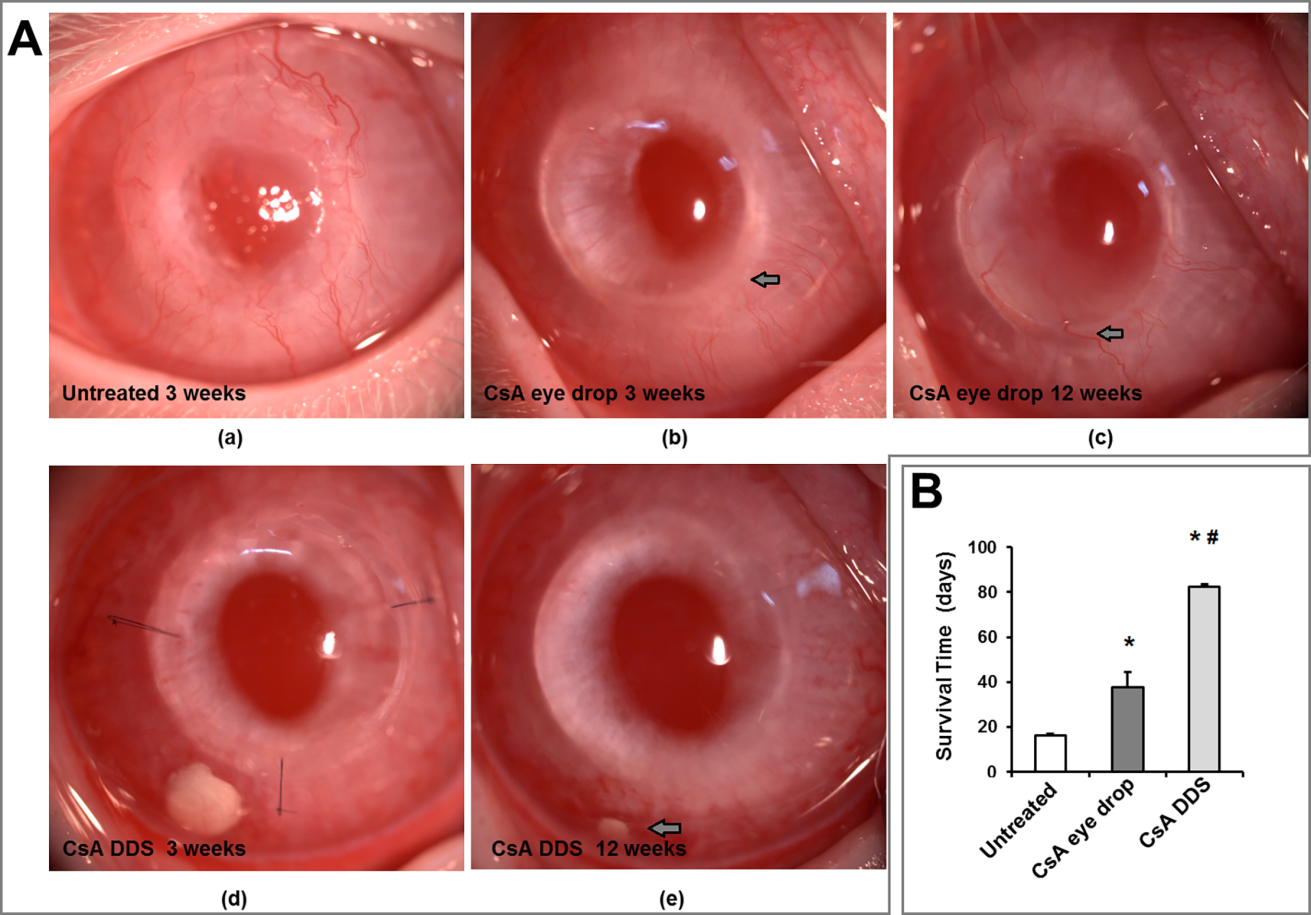
The slit lamp micrographs and corneal graft survival time. (A) Representative slit lamp micrographs showed the corneal status in the untreated, CsA eye drop, and CsA DDS groups at 3 and 12 weeks postoperatively, respectively. (a) Rejected corneal allograft in the untreated group on postoperative week 3, with severe corneal edema and neovascularization. (b-c) Clear corneal allograft with neovasculature invading the graft-host junction (arrow), and mild graft edema with extensive neovasculature invading the graft (arrow) in the CsA eye drop group on postoperative weeks 3 and 12, respectively. (d-e) Clear corneal allografts in the CsA DDS group on postoperative weeks 3 and 12, with the DDS dissolved gradually (arrow). (B) The mean survival time (days) of the corneal allografts in different groups. Data are presented as mean ± SD. * p < 0.01 *vs*. untreated, ^#^ p < 0.01 *vs*. CsA eye drops (n = 10 per group).

The mean graft survival time was 16, 37, and 84 days in the untreated, CsA eye drop and CsA DDS-implanted groups, respectively. In the untreated group, graft rejection occurred within 3 weeks of keratoplasty, with pronounced graft edema, severe opacity, and extensive neovasculature invading the grafts. However, the mean graft survival time in the CsA DDS-implanted group was significantly prolonged compared to the other groups. The corneal allografts treated with CsA DDS remained transparent for more than 12 weeks, with less neovasculature extending into the grafts (Fig 1, S1 Fig).

### Central corneal thickness

**Fig 2:**
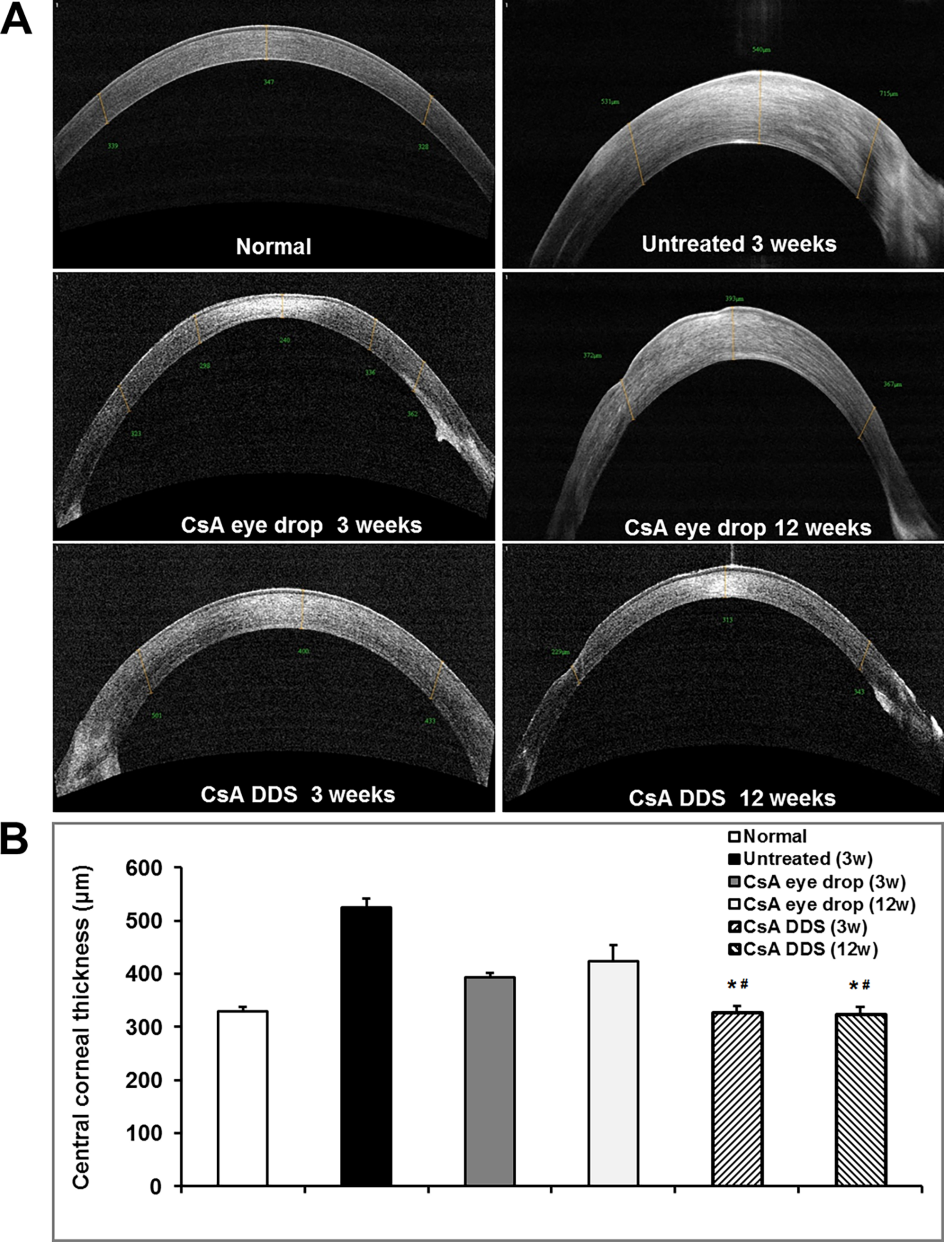
Central corneal thickness analysis. (A) Representative AS-OCT images showed rejected and survived grafts in the untreated, CsA eye drop, and CsA DDS groups. (B) Summarized data depicted reduced central corneal thickness in the CsA DDS group at 3 and 12 weeks postoperatively. The data are depicted as mean ± SD. * p < 0.01 vs. untreated, ^#^ p < 0.01 *vs*. CsA eye drops (n = 6 per group).

As increased corneal thickness has been regarded as a predictor of graft rejection [14, 15], we examined the CCT using AS-OCT at 3 and 12 weeks post-PKP. Compared with the normal control group, CCT in the untreated allografts was significantly increased at 3 weeks (p < 0.01). Compared with the untreated and CsA eye drop treated grafts, CsA DDS treatment gained a lower CCT (all p < 0.01), and there was no difference in CCT between the normal control and CsA DDS groups at all time-points (all p > 0.05) (Fig 2).

### Immunocyte density in the corneal allografts

**Fig 3:**
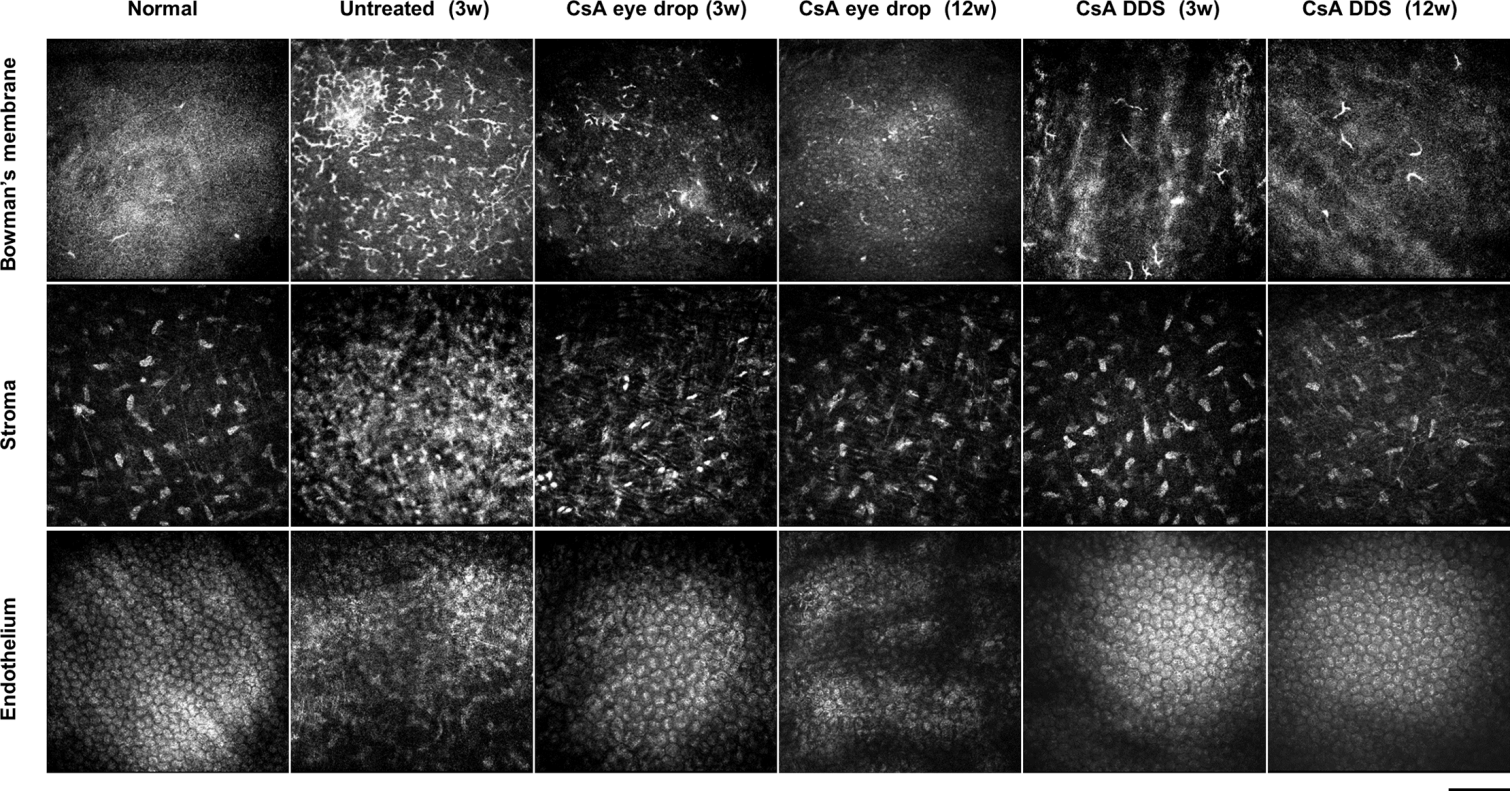
Laser scanning confocal microscopy examination. Representative confocal images of corneal Bowman’s membrane, stroma, and endothelium showed that CsA DDS alleviated inflammatory cell infiltration and maintained a normal endothelial cell density at all time-points (scale bar of 100 μm, n = 6 per group).

Histopathological changes in different layers of the central cornea were evaluated by laser scanning confocal microscopy at 3 and 12 weeks after PKP. Compared with the normal control group, increased accumulation of Langerhans cells in Bowman’s membrane, elevated inflammatory infiltration in the stroma, and a marked decline in endothelial cell density were observed in the untreated allografts at 3 weeks postoperatively (all p < 0.01). At all time-points during the follow-up, the Langerhans and inflammatory cell density in the CsA DDS group was much lower than in the untreated and CsA eye drop groups. Moreover, the endothelial cell density in the CsA DDS group was much higher than the CsA eye drop group at 12 weeks after PKP (Fig 3, Table 2).

**Table 2:** Cell density in different layers of grafts (cells/mm^2^; mean ± standard deviation).

Group	Normal	Untreated 3w	Eye drop 3w	Eye drop 12w	CsA DDS 3w	CsA DDS 12w
Langerhans cells in BM’s layer	18.75 ± 6.25	880.75 ± 110.77	273.75 ± 42.71*	105.00 ± 27.03*	43.75 ± 6.25* ^#^	28.13 ± 8.07* ^#^
Inflammatory cells in the stroma	14.58 ± 3.61	864.58 ± 95.47	175.00 ± 31.25*	166.67 ± 12.84*	22.92 ± 3.61* ^#^	25.00 ± 6.25* ^#^
Endothelial cells	2491.88 ± 126.34	1254.17 ± 56.37	2501.06 ± 143.26*	2123.96 ± 138.77*	2564.06 ± 161.41*	2468.5 ± 165.30* ^#^

BM: Bowman’s membrane

* p< 0.01, versus untreated group;

^#^ p< 0.01, versus CsA eye drop group.

**Fig 4:**
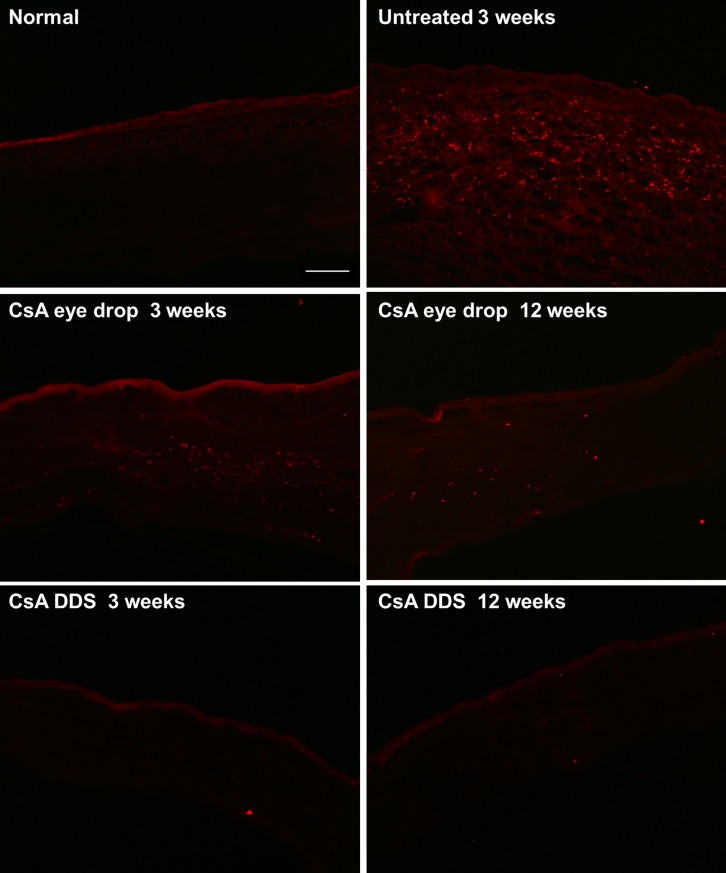
Immunofluorescence photographs of the corneal grafts. Representative images from the central cornea stained with CD4 showed less CD4^+^ T cell infiltration into the grafts in the CsA DDS group compared with the untreated and CsA eye drop groups at all time-points (scale bar of 100 μm).

Previous reports have shown that corneal allograft rejection is a form of CD4^+^ Th1 cells mediated delayed hypersensitivity response [16, 17]. Thus, we counted the CD4^+^ positive T cells, and calculated the density of CD4^+^ T cells in the allografts. Photographs were taken at a distance of 2.5 mm within the central cornea, and two investigators (TZ, TL) counted the CD4^+^ positive T cells in a masked manner. Our results demonstrated that the CD4^+^ T cell density in the corneal allograft was 7.50 ± 2.80, 707.50 ± 54.98, 160.16 ± 63.99, and 8.75 ± 3.06 cells/mm^2^ in the normal control, untreated, CsA eye drop, and CsA DDS groups at 3 weeks, respectively. The CD4^+^ T cell density was 66.67 ± 10.94 and 10.00 ± 3.42 cells/mm^2^ in the CsA eye drop and CsA DDS groups at 12 weeks, respectively (Fig 4). Compared with the normal control group, an increased CD4^+^ T cell density was observed in the untreated allografts at 3 weeks (p < 0.01). At all time-points during the follow-up, CsA DDS treatment significantly reduced the CD4^+^ T cell infiltration (all p < 0.01 *vs*. the untreated and CsA eye drop groups). However, CD4^+^ T cells are composed of regulatory T cells benefiting graft survival and effector T cells harming graft survival. Therefore, qRT-PCR analysis of interferon-gamma (IFN-γ) and forkhead box p3 (FOXP3) were performed to further detect the type of CD4^+^ T cells infiltrated in the grafts. Moreover, immunofluorescence staining depicted less CD11b^+^ and CD8^+^ cell infiltration in the CsA DDS group compared with the untreated and CsA eye drops treated allografts at all time-points (S2 Fig and S3 Fig).

### Expression of mRNA in the corneal allograft

**Fig 5:**
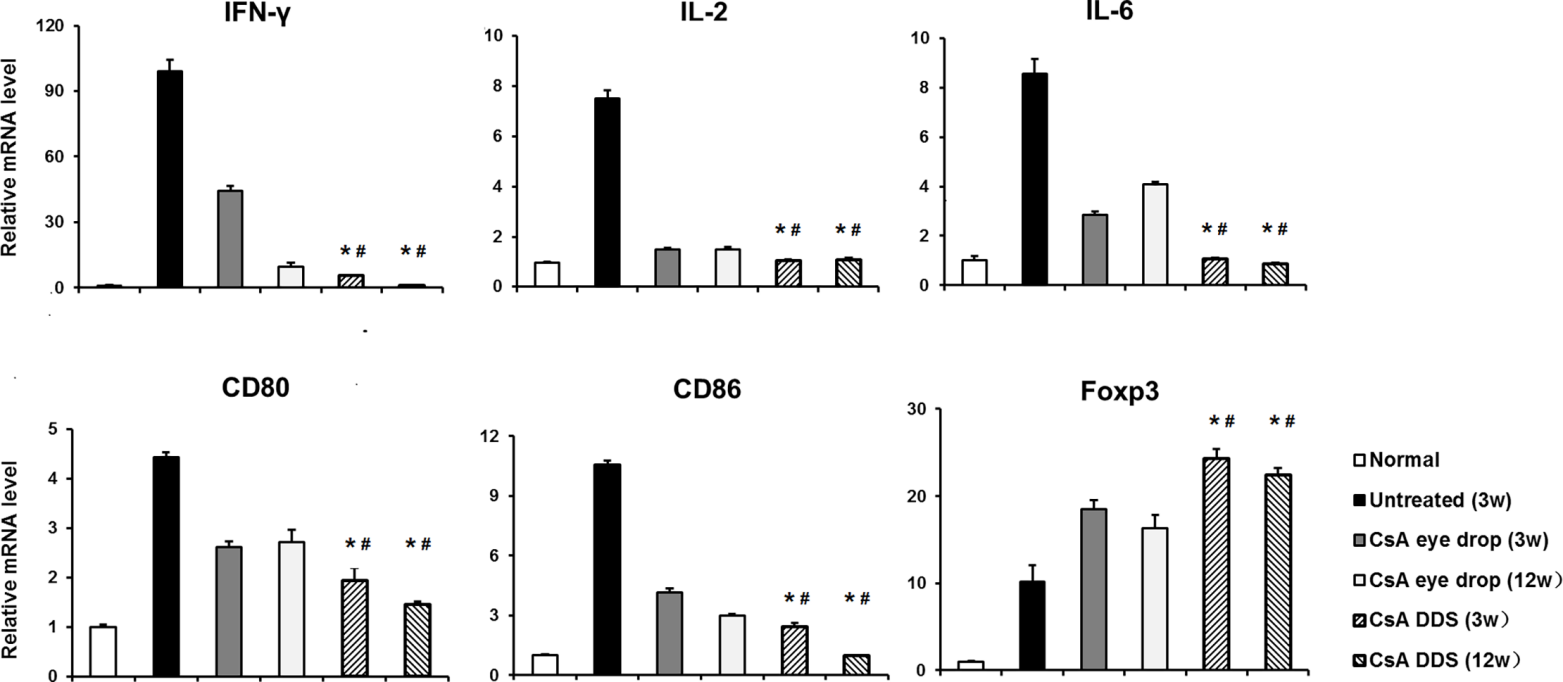
The expression of mRNA in the grafts. Compared with the untreated and CsA eye drop groups, the mRNA levels of IFN-γ, IL-2, IL-6, CD80, and CD86 were significantly decreased, while the transcriptional level of FOXP3 was significantly increased in the CsA DDS group at 3 and 12 weeks postoperatively. The data are presented as mean ± SD. * p < 0.01, *vs*. untreated, ^#^ p < 0.01 *vs*. CsA eye drops (n = 4 per group).

QRT-PCR revealed a marked up-regulation of pro-inflammatory cytokines in the untreated grafts, including IFN-γ, IL-2, and IL-6, as well as co-stimulatory molecules CD80 and CD86 (all p < 0.01). In the groups treated with CsA eye drops and CsA DDS, the mRNA levels of pro-inflammatory cytokines and co-stimulatory molecules decreased significantly with the follow-up time. However, the expression of pro-inflammatory cytokines and co-stimulatory molecules in the CsA DDS-implanted corneal allografts was more significantly down-regulated than the CsA eye drops counterparts (all p < 0.01). The results also demonstrated that the mRNA level of IFN-γ was increased more significantly than the FOXP3 in the untreated grafts. However, the mRNA level of FOXP3 was increased more significantly than the IFN-γ in the CsA treated groups (Fig 5).

The clinical evaluation, histopathological examination, and qRT-PCR analysis demonstrated that, compared with the untreated and CsA eye drop groups, CsA DDS implantation decreased inflammatory cell infiltration and pro-inflammatory cytokine levels, and maintained a normal CCT.

### CD4^+^ T cell density and mRNA expression in the iris-ciliary body

Since the iris-ciliary body and anterior chamber were reported to be involved in the pathogenesis of corneal allograft rejection [18, 19], we investigated the influence of CsA DDS treatment on the iris-ciliary body and aqueous humor.

**Fig 6:**
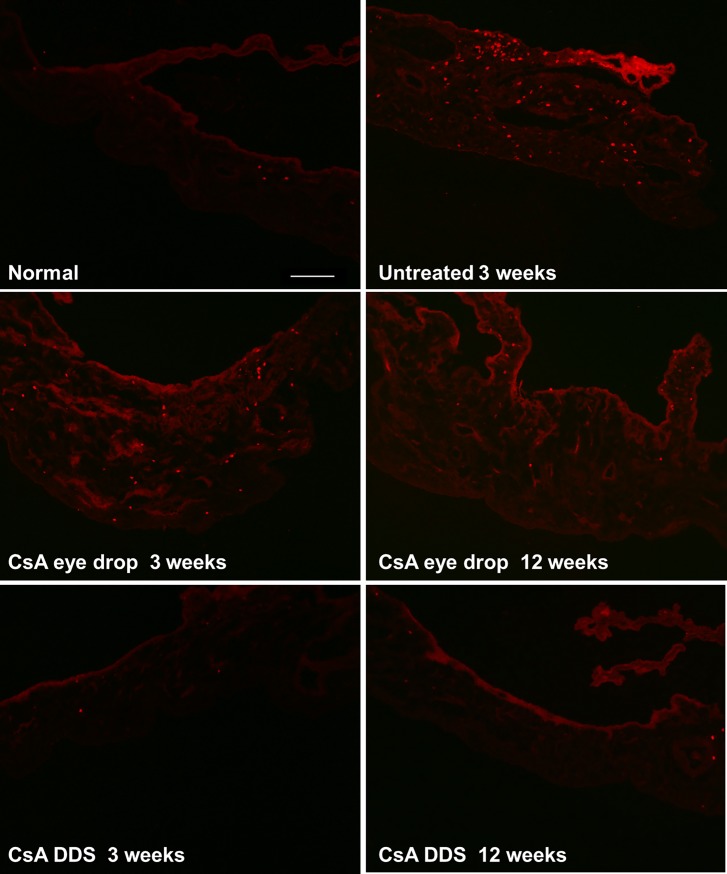
The immunofluorescence photographs of iris-ciliary body. s-ciliary bodies showed less CD4^+^ T cell infiltration at all time-points (scale bar of 100 μm).

The CD4^+^ T cell density in the iris-ciliary body was found to be 35.42 ± 3.61, 498.75 ± 44.72, 187.50 ± 47.39, and 31.25 ± 6.25 cells/mm^2^ in the normal control, untreated, CsA eye drop group, and CsA DDS groups at 3 weeks, respectively. The CD4^+^ T cell density was 110.42 ± 41.21 and 29.17 ± 7.22 cells/mm^2^ in the CsA eye drop and CsA DDS groups at 12 weeks, respectively (Fig 6). Compared with the normal control group, an increased CD4^+^ T cell density was observed in the untreated iris-ciliary body at 3 weeks (p < 0.01). At all time-points during the follow-up, CsA DDS treatment significantly reduced the CD4^+^ T cell infiltration (all p < 0.01 *vs*. the untreated and CsA eye drop groups).

**Fig 7:**
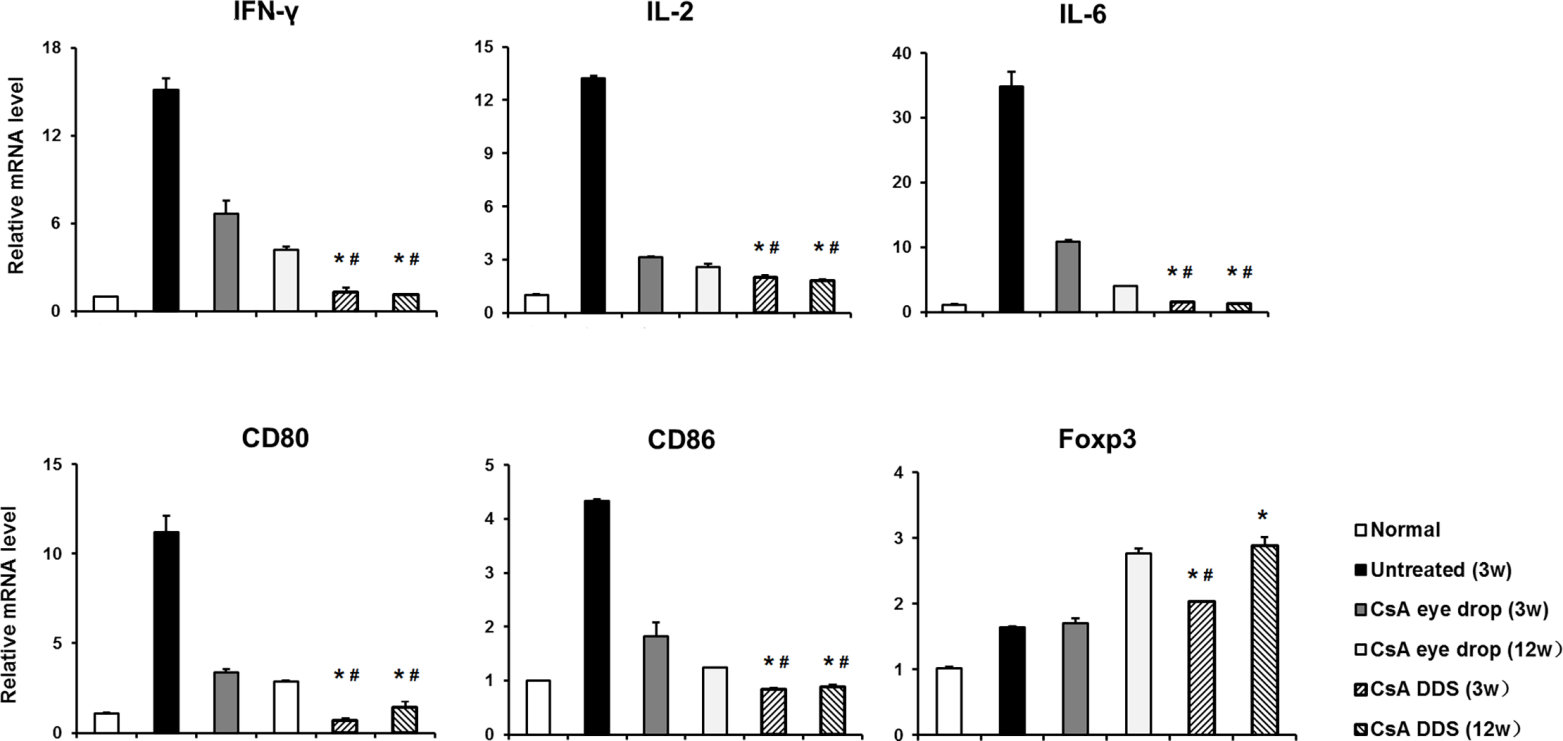
The expression of mRNA in the iris-ciliary body. Compared with the untreated and CsA eye drop groups, the mRNA levels of IFN-γ, IL-2, IL-6, CD80, and CD86 were significantly decreased, while the transcriptional level of FOXP3 was increased in the CsA DDS group at all time-points. The data are presented as mean ± SD. * p < 0.01, vs. untreated, ^#^ p < 0.01 vs. CsA eye drops (n = 4 per group).

We also tested the expression of pro-inflammatory cytokines and co-stimulatory molecules in the iris-ciliary body. The results demonstrated elevated expressions of IFN-γ, IL-2, IL-6, CD80, and CD86 in the untreated iris-ciliary body (all p < 0.01). However, the expression of cytokines and co-stimulatory molecules in the CsA DDS-treated iris-ciliary bodies was significantly down-regulated than in the untreated and CsA eye drop-treated ones (all p < 0.01). The results also showed that the mRNA level of IFN-γ was increased more significantly than the FOXP3 in the untreated iris-ciliary body. However, the mRNA level of FOXP3 was increased more significantly than the IFN-γ in the CsA treated iris-ciliary bodies (Fig 7). CsA DDS blocked the immune response that occurred in the iris-ciliary body during corneal allograft rejection.

### IL-2 concentration in the aqueous humor

**Fig 8:**
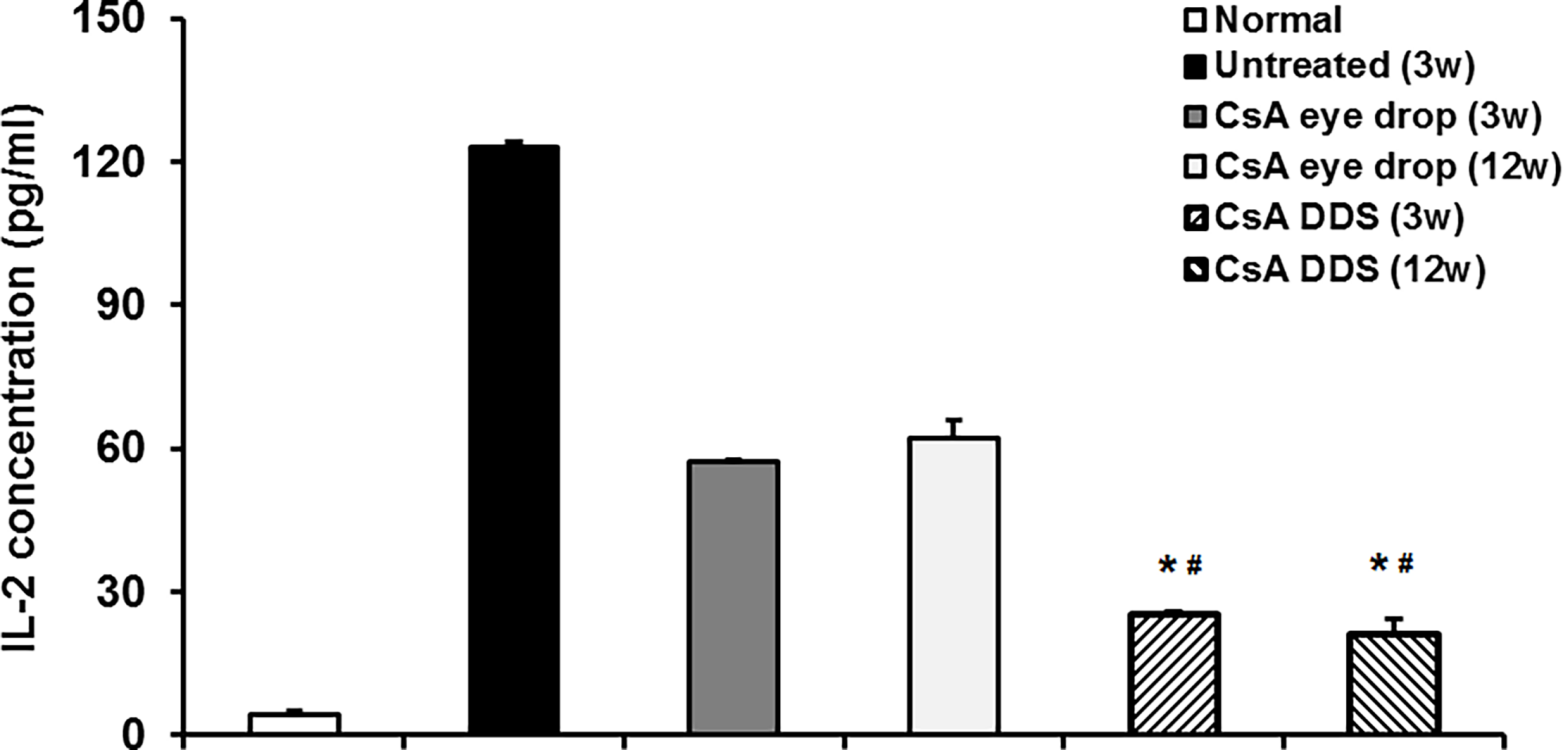
IL-2 level in the aqueous humor. ELISA test demonstrated that IL-2 concentration was significantly increased in the untreated group at 3 weeks, and CsA DDS treatment more effectively decreased the level of IL-2 in the aqueous humors at all time-points. The data are presented as mean ± SD. * p < 0.01 *vs*. untreated, ^#^ p < 0.01 *vs*. CsA eye drops (n = 6 per group).

IL-2 is a representative pro-inflammatory cytokine that reinforces the immune response [20, 21]. Accordingly, we measured the IL-2 concentration in the aqueous humor, which was 4.41± 0.53, 123.21 ± 0.91, 57.34 ± 0.33, and 25.09 ± 0.64 pg/ml in the normal control, untreated, CsA eye drop, and CsA DDS groups at 3 weeks, respectively. The IL-2 concentration was 62.04 ± 4.01 and 21.10 ± 3.21 pg/ml in the CsA eye drop and CsA DDS groups at 12 weeks, respectively. Compared with the control group, the IL-2 level in the untreated group was significantly increased (p < 0.01). However, the IL-2 level was markedly decreased after CsA treatments (p < 0.01), and, in particular, was much lower in the CsA DDS treated group (p < 0.01) (Fig 8). This may partially explain the superiority of CsA DDS over eye drops in the prophylaxis of corneal allograft rejection.

## Discussion

CsA DDS could effectively prevent corneal allograft rejection in animal models and clinical trials [11, 12], although the underlying mechanisms remain largely unknown. In the present study, we found that CsA DDS treatment not only alleviated the immune response in corneal allograft, but also significantly improved the intraocular immune microenvironment, including the iris-ciliary body and aqueous humor.

It is well established that the cornea benefits from anatomical advantages of immune privilege [22]. Aqueous humor and iris-ciliary body pigment epithelial cells contain various immunosuppressive factors that subdue the effector functions of immune cells, and these elements may induce the formation of anterior chamber associated immune deviation (ACAID) [23, 24]. However, ACAID can be easily disturbed by postoperative inflammation, neovascularization, and trauma, suffering the cornea and the endothelial cells as direct targets of immune attacks [25].

In our study, when rejection occurred, the CD4^+^ T cell density, the transcriptional levels of IFN-γ, IL-2, IL-6, CD80, and CD86 in both the corneal graft and the iris-ciliary body, as well as the protein level of IL-2 in the aqueous humor, were prominently increased. The results of our study agree with previous investigations which revealed when rejection occurred, an increasing number of immune cells emerged in the iris-ciliary body and anterior chamber, similar to the pathological changes that occurred in the corneal allograft [18, 19, 26]. Results in this study, combined with the findings of inflammatory cell aggregation on the endothelium and the observation of immune cell and cytokine infiltration in the aqueous humor of recipients with allograft rejection [27–31], suggest the possibility of iris-ciliary body as an origin of inflammatory cells, and the involvement of the intraocular pathway in the pathogenesis of corneal graft rejection.

We utilized the laser scanning confocal microscope to analyze the Langerhans and inflammatory cell density [32]. Because of its high sensitivity and good reproducibility, confocal microscopy has been widely used to investigate the corneal status. CsA DDS was observed to be more helpful in alleviating Langerhans and inflammatory cell aggregation and sustaining a physiological endothelial cell density than CsA eye drops. Furthermore, the immunofluorescence staining depicted less CD4^+^, CD11b^+^, and CD8^+^ cell infiltration in the grafts treated with CsA DDS than those with CsA eye drops.

In the current study, most infiltrated CD4^+^ T cells were found to be effector T cells during corneal allograft rejection by qRT-PCR. CsA treatments decreased the number of effector CD4^+^ T cells, and increased the population of regulatory T cells in the graft and iris-ciliary body, which was consistent with those reported in previous studies [33–35]. However, the influence of CsA on the regulatory T cells has remained controversial. In contrast to our results, recent studies showed that CsA inhibited tolerance induction and even abrogated established tolerance [36, 37]. It was expected that CsA interfered the induction and the function of regulatory T cells at high concentrations [38]. Low-dose CsA, however, showed positive and cumulative effects on tolerance in different transplantation models [33–35]. The specific effects of CsA on regulatory T cells may depend on its concentration. We hypothesize that the application of CsA eye drops or CsA DDS could gain a relatively low drug concentration in the cornea and iris-ciliary body, which might induce immune tolerance.

The superiority of CsA DDS among the experimental groups can be explained as follows. First, CsA DDS more significantly prolonged the graft survival time. Second, CsA DDS more markedly decreased inflammatory cell infiltration and pro-inflammatory cytokine expression in the corneal graft, iris-ciliary body, and aqueous humor. Third, CsA DDS treatment was more effective in maintaining a normal CCT and sustaining healthy endothelial cells. Therefore, it is reasonable to assume that implantation of CsA DDS in the anterior chamber is a promising strategy for the prevention of high-risk corneal allograft rejection.

CsA DDS not only significantly attenuated the immune response in corneal allograft, but also pronouncedly alleviated the immune response in the iris-ciliary body and aqueous humor. Given that the iris-ciliary body might be an origin of inflammatory cells which cause endothelial rejection, we speculate that CsA DSS implanted in the anterior chamber could significantly block the egression of inflammatory cells from the iris-ciliary body that target to the endothelium.

Corneal allograft rejection is a complex procedure predominantly mediated by T cells and regulated by numerous cytokines. CsA could reduce the secretion of various lymphokines, including IL-2, through down-regulating the translocation of the nuclear factor of activated T cells (NFAT) [39–41]. The increased level of IL-2 in the aqueous humor might be responsible for the induction of immune reaction [42]. Our results showed that CsA DDS treatment significantly decreased the transcriptional levels of IFN-γ, IL-2, and IL-6 in the corneal allograft and the iris-ciliary body, as well as the protein level of IL-2 in the aqueous humor. However, whether this process was related to the NFAT requires further investigation.

The sensitization of allogeneic T cells requires two signals. The first signal is provided by T cell receptor-CD3 recognition of the major histocompatibility complex (MHC), a peptide complex presented by the antigen-presenting cells (APCs); the second signal is provided by the interaction between CD80 and CD86 on the APCs with its receptor CD28 on the T cells [43–46]. The inflammatory milieu caused by corneal transplantation results in high levels of MHCⅡ and co-stimulatory molecules, which prime naïve CD4^+^ T cells into Th1 effectors, the principal mediators of acute corneal graft rejection [20, 21].

CsA DDS treatment decreased the Langerhans and CD11b^+^ cell infiltration into the allografts in this study, which represents the main APCs participating in the corneal allograft rejection. Meanwhile, CsA DDS also down-regulated the transcriptional level of CD80 and CD86 both in the corneal graft and iris-ciliary body, and accumulated evidence indicates that inhibiting the CD80/CD86 pathway is effective in the prevention of corneal allograft rejection and autoimmune anterior uveitis [47–49]. The results suggest that CsA DDS implantation may be a potential mean of inducing immune tolerance in high-risk corneal transplantation. The low levels of CD80 and CD86 in the CsA DDS group may represent a weaker immune response, while the higher levels of the same targets in the untreated and CsA eye drop groups may contribute to an earlier allograft rejection.

## Conclusions

Intraocular implantation of CsA DDS seems to be effective in the prevention of immune rejection after keratoplasty, and the mechanism involved is probably related to its comprehensive improvement of the immune microenvironment in the corneal allograft, iris-ciliary body, and aqueous humor.

## Supporting information

S1 Fig Representative slit lamp micrographs in different groups. (TIF)

S2 Fig Immunofluorescence photographs of the corneal graft (CD11b, scale bar of 50 μm). (TIF)

S3 Fig Immunofluorescence photographs of the corneal graft (CD8, scale bar of 100 μm). (TIF)
