# Supplementary figures and images for: Cyclosporine a drug-delivery system for high-risk penetrating keratoplasty: Stabilizing the intraocular immune microenvironment
Source: PLoS One. 2018 May 7;13(5):e0196571. doi: 10.1371/journal.pone.0196571 (PMC5937766; doi:10.1371/journal.pone.0196571)

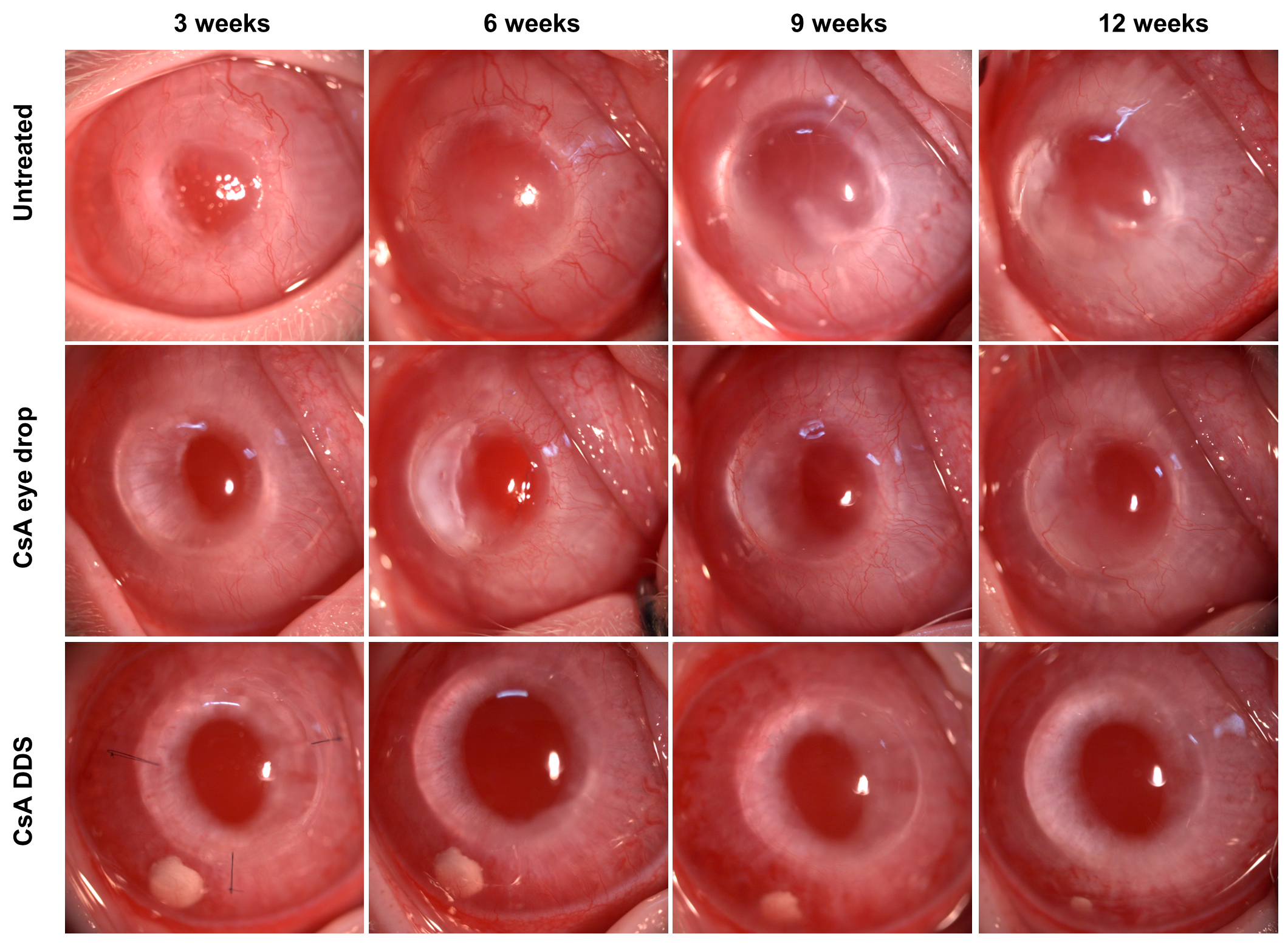

Supplement: S1 Fig — (TIF) [file pone.0196571.s001.tif]

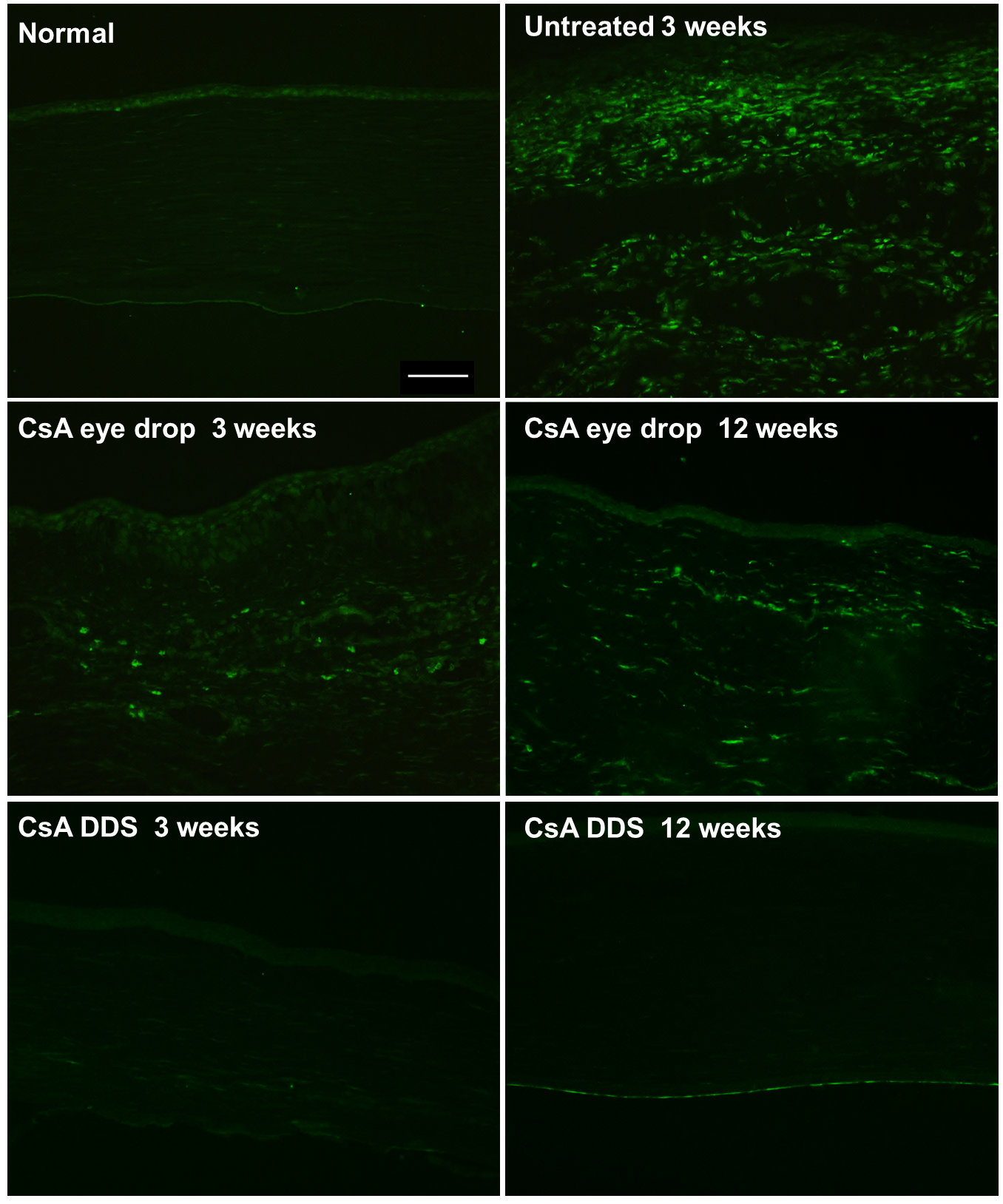

Supplement: S2 Fig — (TIF) [file pone.0196571.s002.tif]

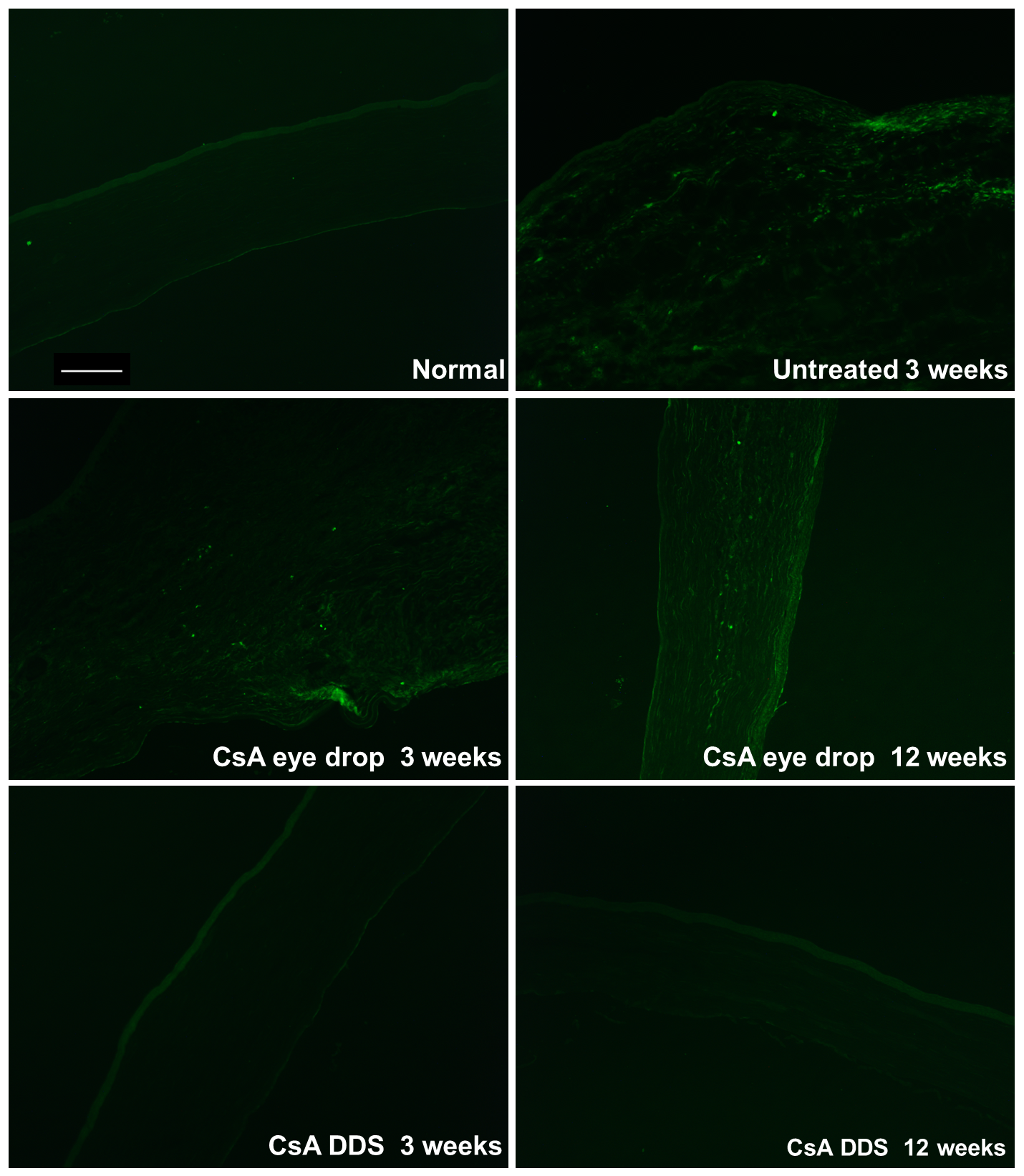

Supplement: S3 Fig — (TIF) [file pone.0196571.s003.tif]
